# Evidence of very low hepatitis B virus prevalence in children and adolescents in Germany: National cross-sectional study, 2014–2017

**DOI:** 10.1017/S0950268825100563

**Published:** 2025-09-26

**Authors:** Sofie Gillesberg Lassen, Christina Poethko-Müller, Martin Schlaud, Heiko Slanina, Christian G. Schüttler, Klaus Stark, Viviane Bremer, Thomas Harder, Sandra Dudareva

**Affiliations:** 1 Department of Infectious Disease Epidemiology, Robert Koch Institute, Berlin, Germany; 2 Charité – Universitätsmedizin Berlin, Berlin, Germany; 3 Department of Epidemiology and Health Monitoring, Robert Koch Institute, Berlin, Germany; 4 Institute of Medical Virology, National Reference Center for Hepatitis B Viruses and Hepatitis D Viruses, German Center for Infection Research (DZIF, Partner Site Giessen-Marburg-Langen), Justus Liebig University, Giessen, Germany; 5 Institute of Public Health, Riga Stradins University, Riga, Latvia

**Keywords:** Hepatitis B virus infection, children and adolescents, anti-HBc, HBs-antigen, Germany, sero-surveys, prevalence

## Abstract

Attaining the target of <0.1% HBsAg positives in children aged <5 years in vaccinated populations by 2030 is a WHO indicator of hepatitis B elimination. We aimed to calculate the prevalence of HBsAg- and anti-HBc-positive children and adolescents in the low-prevalence country of Germany. In total, 3567 children and adolescents aged 3–17 years participated in a national population based cross-sectional study. Data were collected between 2014 and 2017 using questionnaires and health examinations, including blood samples. Applying a weighted analysis to account for survey design and participant characteristics, we calculated the HBsAg and anti-HBc prevalence and described them by anti-HBs positivity. In total, 3007 participants had all three sero-markers measured. None were found HBsAg and anti-HBc positive. Seven (0.3%, 95% CI: 0.1–0.8) were anti-HBc positive and HBsAg negative; six were also anti-HBs positive. All anti-HBc-positive participants were aged ≥7 years and three had no migration background. Four anti-HBc-positive participants had known vaccination status; three had been vaccinated according to national recommendations. This very low hepatitis B virus sero-prevalence among children and adolescents indicates that Germany is reaching some hepatitis B virus elimination targets. We recommend maintaining preventive measures, in particular a high vaccination coverage, in order to reach hepatitis B elimination.

## Introduction

A perinatal infection with hepatitis B virus (HBV) leads to chronic disease in 80–90% of infants if no post-exposure prophylaxis is given [1]. According to WHO, in 2022, an estimated 1.23 million (0.81–1.53 million) people globally were newly infected with HBV, and 1.10 million (0.88–1.74 million) people died from HBV infection related causes [2, 3]. In 2016, WHO published a global strategy to eliminate viral hepatitis, including hepatitis B, as a public health threat by 2030 [4]. The WHO European region followed with an action plan for hepatitis elimination in 2017 [5]. In 2020, a framework for action for Intersectoral Collaboration to end HIV, tuberculosis, and viral hepatitis in Europe and Central Asia was published [6]. In 2022, WHO published global strategies for 2022–2030, including updated indicator targets [7]. Global targets for HBsAg prevalence in 0- to 4-year-olds was set at ≤0.5% in 2025 and ≤0.1% in 2030 as proof of reaching elimination of mother-to-child transmission of HBV [5–8]. In Germany, prevention of mother-to-child transmission strategies include universal hepatitis B childhood vaccination, introduced in 1995; and hepatitis B screening during pregnancy, introduced in 1994, concurrently with post-exposure prophylaxis consisting of passive and active hepatitis B immunization for children born to a HBV-positive person or a person with unknown HBV status [9, 10]. According to school health entry examination data for children born between 2012 and 2015, HBV vaccination coverage was 87.3% with a range of 80.4–93.7% in the different federal states [11]. According to health insurance data, 79.1% of children born in 2019 had been given a complete HBV vaccination series at 24 months of age [11].

Previous studies have shown that Germany has a low prevalence of HBV in the general population, with higher prevalence observed among groups with higher risk of infection, such as people injecting drugs, migrants from high-prevalence countries, and men who have sex with men [12–15]. Prevalence surveys using three serological markers – HBsAg, anti-HBc, and anti-HBs – can be used to assess the epidemiological situation of HBV in different population groups. In Germany, a national population based survey conducted in adults in 2008–2011 estimated an HBsAg prevalence of 0.3% and an anti-HBc prevalence of 5.1% [14]. A scoping review found that estimates of the overall prevalence of HBsAg in the general adult population in Germany were the lowest in the national population based survey mentioned earlier [14], and ranged to 1.6% in proxy populations used to assess the overall prevalence in the general population [12]. Estimates from proxy populations were higher and had higher uncertainties [12]. In groups with increased risk of HBV infection, such as people injecting drugs, migrants from high prevalence countries, and men who have sex with men, HBsAg prevalence ranged from 0.2% to 4.5% [12].

To date, HBV sero-prevalence has been assessed only once in a population based sample of children and adolescents in Germany. The German Health Interview and Examination Survey, KiGGS Baseline, a population based survey conducted in 2003–2006, found 0.5% (95% confidence interval (CI): 0.4–0.7) of 3- to 17-year-olds to be anti-HBc positive and 38.7% (95% CI: 20.0–57.5) of the anti-HBc positives to also be HBsAg positive [15].

Using cross-sectional data collected during the second wave (2014–2017) of the population based German Health Interview and Examination Survey, KiGGS Wave 2, the objectives of this study were as to calculate the prevalence of hepatitis B sero-markers (HBsAg and anti-HBc) and to describe combinations of sero-markers (HBsAg, anti-HBc and anti-HBs) in children and adolescents aged 3–17 years in Germany by demographic and health characteristics. The findings are used to assess the status of the elimination of HBV in Germany and to inform further control and elimination measures.

## Methods

### Study population, KiGGS Wave 2

KiGGS Wave 2 data were collected in 2014–2017 at 167 sampling points across Germany. Sampling points were selected in order to have a nationally representative cross-sectional sample of 0- to 17-year-olds. A total of 3567 children and adolescents aged 3–17 years were part of a randomly allocated sample that underwent physical examinations and completed self-administered questionnaires. The response rate was 41.5%. As part of the examinations, blood samples and copies of vaccination records were collected. Sampling strategy, data collection, and representativeness were previously described by Mauz et al. [16], Hoffmann et al. [17], and Frank et al. [18]. As the blood samples were collected to examine a range of serological parameters including HBV sero-prevalence, we calculated a margin of error [19] expecting to have reached the WHO target of <0.1% HBsAg positives as well as a lower anti-HBc prevalence than the KiGGS Baseline results [15]. For an expected prevalence of 0.09% for HBsAg and 0.3% for anti-HBc, this sample size (3567) would allow calculation of prevalence estimates with a margin of error of 0.0026 for HBsAg and 0.00586 for anti-HBc.

### Serological testing

Serum samples were analysed for HBsAg (HBsAG Qualitative II, Limit of detection (LoD) 0.02 IU/mL), anti-HBc IgG (anti-HBc II, LoD 0.5 PEI U/mL), and anti-HBs IgG (anti-HBs, LoD 0.98 IU/L) using the commercially available microparticle chemiluminescence ARCHITECT System (Abbott, Illinois, USA) with the appropriate assays and reagents according to the manufacture’s specifications. The manufacturer reports the following sensitivities and specificities: Anti-HBc II sensitivity of 100% (95% CI: 99.1–100, analytical sensitivity of 0.4–0.5 PEI U/mL), overall specificity of ≥99.5% (16); anti-HBs overall sensitivity of 97.54% (95% CI: 95.97–98.62), and overall specificity of 99.67% (95% CI: 99.22–99.89) [17]. The limit of blank was 0.5 mIU/mL, the limit of detection 0.98 mIU/mL, and the quantitation range was 2.50–1000.00 mIU/mL. Samples with anti-HBs levels exceeding 1000 mIU/mL were automatically diluted and re-analysed. The HBsAg Qualitative II had a reported sensitivity of 99.93% (95% CI: 99.62–100), an analytical sensitivity of 0.017–0.022 IU/mL, and a specificity of 99.91% (95% CI: 99.78–99.97) in blood donors.

Serum samples with a level of anti-HBs exceeding 1000 IU/L were automatically diluted and re-assayed. Quality control specimens on HBsAg (positive, negative), anti-HBc IgG (positive, negative), and anti-HBs IgG (low, medium, high) were measured before and after each daily run of serum analyses. The Central Epidemiological Laboratory passed all 14 round-robin tests on the mentioned serological markers, issued by Instand (Instand e.V., Düsseldorf, Germany) and RfB (RfB Referenzinstitut für Bioanalytik, Bonn, Germany) during the time of study.

HBsAg levels ≥1 S/CO, anti-HBc levels ≥1 S/CO, and anti-HBs levels ≥10 mIU/mL (by anti-HBc negatives) were defined as positive. Samples that tested anti-HBc positive, but both HBsAg and anti-HBs negative were re-tested in-house. All HBsAg-positive samples were sent to the University of Essen, Germany, for PCR analysis.

In addition, all samples that tested positive for anti-HBc and/or HBsAg underwent a second evaluation at the National Reference Centre for Hepatitis B Viruses and Hepatitis D Viruses. In cases where the anti-HBc results may have been false positive, an anti-HBc neutralization test was employed for verification purposes. Re-evaluated samples with an Anti-HBs level between 2 and <10 mIU/mL were redefined as anti-HBs positive. Samples were only regarded HBsAg positive when confirmed at the National Reference Laboratory.

**Figure 1.**
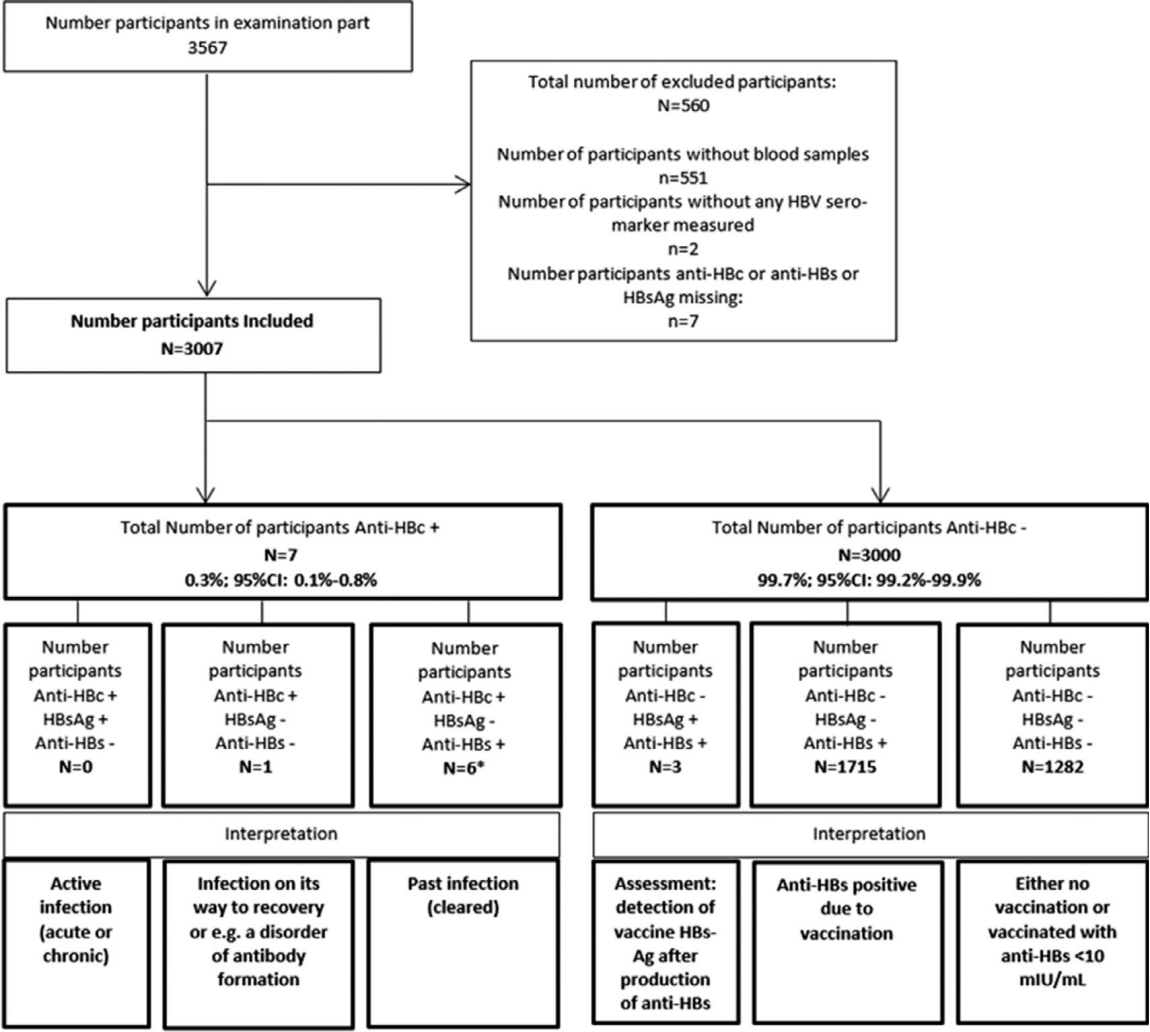
Flowchart of participants included and excluded including the number and weighted proportions of anti-HBc positives and negative participants and their 95% confidence intervals, as well as the number of participants by HBV sero-marker combinations and their interpretation.

The sero-marker combination of HBsAg positive, anti-HBc positive, and anti-HBs negative indicates an active acute or chronic infection. A sero-marker combination of anti-HBc positive, but HBsAg and anti-HBs negative, can indicate either an infection on its way to recovery or, for example, a disorder of antibody formation (Figure 1). For all other sero-marker combinations with positive HBsAg or anti-HBc, the National Reference Centre provided expert opinion for interpretation.

### Statistical analysis

We estimated the HBsAg and anti-HBc prevalence and their 95% confidence intervals (CI) using a weighted proportion. Among anti-HBc negatives, we also estimated the prevalence of anti-HBs positives and negatives. The weighting took both the survey design and participation rate into account. The weighting corrected the estimate according to the German Micro Census from 2013 with regard to age (years), sex, federal state, German citizenship, and educational level of parents using the classification of the Comparative Analysis of Social Mobility in Industrial Nations [20, 21]. We calculated the coefficient of variation (CV) for the anti-HBc prevalence estimate. In the absence of observed positives, a one-sided 95% upper confidence bound was calculated using the Clopper–Pearson exact method.

We included participants with all three HBV sero-markers measured in our primary analysis. In a sub-analysis for comparison with the estimates from KiGGS Baseline, we included 3012 participants from KiGGS Wave 2 and 13062 participants from KiGGS Baseline aged 3–17 years that had both anti-HBc and anti-HBs measured. This corresponds to the serological testing conducted for participants during KiGGS Baseline and age of tested in KiGGS Wave 2. For the primary analysis, we compared demographic characteristics included participants with the complete study population. For categorical variables, we calculated frequencies and proportions. For continuous variables, we calculated medians and interquartile ranges (IQR).

HBsAg- and anti-HBc-positive participants were described according to the following demographic and health characteristics: sex (male, female); age group at time of study (3–6, 7–10, 11–13, 14–17); place of living (eastern or western Germany); municipality size (small, medium-sized, large town, or urban area); migrant background (none, one-sided, two-sided); and participation in voluntary recommended early childhood development visits at their paediatrician [22]. Regarding hepatitis B vaccination status, we described the number of doses and the time interval between the third and fourth doses. Additionally, weighted univariable logistic regression analyses were conducted for demographic and health characteristics. All analyses were conducted using STATA 17.0 (StataCorp).

## Results

A total of 3007 (84%) out of 3567 participants had all three serological markers measured (Figure 1). Their median age was 11 years (IQR: 7–14 years) and 49% were male. Two-thirds (66%) were living in West Germany and 23% had a migration background (Table 1).

**Table 1.** Characteristics of included participants, KiGGS Wave 2, N = 3007

Characteristics		*n* (%)
Sex	Male	1485 (49)
	Female	1522 (51)
Age group (years)	3–6	625 (21)
	7–10	744 (25)
	11–13	733 (24)
	14–17	905 (30)
Birth cohort	1994–1997	62 (2)
	1998–2001	864 (29)
	2002–2005	901 (30)
	2006–2009	736 (24)
	2010–2013	442 (15)
	2014–2017	2 (0.1)
Geographical place of living	East Germany (including Berlin)	1021 (34)
	West Germany	1986 (66)
Municipality size	<5000 inhabitants (rural)	617 (21)
	5000 < 20000 (small town)	864 (29)
	20000 < 100000 inhabitants (medium-sized town)	839 (28)
	≥100000 inhabitants (Urban)	687 (23)
Socio-economic status	Low (1st quintile)	425 (14)
	Middle (2nd quintile)	578 (19)
	Middle (3rd quintile)	553 (18)
	Middle (4th quintile)	652 (22)
	High (5th quintile)	696 (23)
	Missing	103 (3.4)
Migration status	None	2244 (75)
	One sided	266 (8.9)
	Two sided	412 (14)
	Missing	85 (2.8)
Migration generation	First generation	94 (3.1)
	Second or more generations	584 (19)
	Missing or non-applicable	2329 (77)
Level of education, CASMIN classification ^[^^21^ ^]^	Low level	319 (11)
	Middle level	1519 (51)
	Higher level	1049 (35)
	Missing	120 (4.0)

Seven of the 3007 included participants were found anti-HBc positive corresponding to a prevalence of 0.3% (95% CI: 0.1–0.8; CV: 49%). All anti-HBc-positive participants were HBsAg negative (0.00%, one-sided 95% upper bound = 0.10%). After re-testing of anti-HBc-positive samples, one anti-HBc-positive sample was anti-HBs negative (<2.00 mIU/mL), three were anti-HBs positive at 2.00 < 10.00 mIU/mL and three were anti-HBs positive at ≥10 mIU/mL (Figure 1).

Of the 3000 (99.7%; 95% CI: 99.2–99.9) anti-HBc-negative participants, 1718 (58.5%; 95% CI: 56.4–60.6) were anti-HBs positive and 1282 (41.2%; 95% CI: 39.1–43.3) were anti-HBs negative. Three anti-HBc-negative participants were found HBsAg and anti-HBs positive, also after re-testing of HBsAg-positive samples (Figure 1). All three were also found PCR negative.

In our sub-analysis, comparing the anti-HBc prevalence estimated here with the prevalence of anti-HBc in KiGGS Baseline (2003–2006), we found similar prevalence with overlapping 95% CI: KiGGS Baseline 0.5% (95% CI: 0.4–0.6) and 0.3% (95% CI: 0.1–0.8) for KiGGS Wave 2. However, the KiGGS Wave 2 estimate had more variation relative to the mean (CV: 49%) than the estimates from KiGGS Baseline (CV: 15%) (Supplementary Table S1).

Of the 560 (16%) participants excluded from the analysis, 551 had no blood samples taken, two did not have any HBV serological markers measured, and seven had one or two HBV serological markers measured. Five of the seven participants with one or two HBV serological markers measured were found anti-HBc negative and the remaining two had no anti-HBc measured. The six participants who had anti-HBs measured were all found positive. The one participant who had HBsAg measured was found negative (Figure 1).

With a mean age of 11 years (IQR: 7–14) and a slightly lower proportion of included participants being 3–6 years old (21%), included participants were slightly older than the complete survey sample. In regard to sex, place of living (geographical and municipality size), socio-economic status, migration background, and level of education, we saw similar proportions within included and overall participants; see Supplementary Table S2.

### Description of anti-HBc-positive participants

The seven anti-HBc-positive and HBsAg-negative participants were aged 7–17 years old, and four were female. Six lived in western federal states, four lived in cities with ≥100000 inhabitants, two lived in medium-sized cities (20000 < 100000 inhabitants), and one lived in a small town (5000 < 20000 inhabitants).

For six of the seven anti-HBc-positive participants, information on migrant status and healthcare utilization was available. Three had no migration background, one had a one-sided migration background (low-endemicity country, categorisation based on https://cdafound.org/polaris/ estimate of HBsAG prevalence among adults) and two had a double-sided migration background (intermediate-endemicity countries, categorisation based on https://cdafound.org/polaris/ estimate of HBsAG prevalence among adults) (Table 2). All six had participated in all recommended health examinations in the first 12 months of life (U1–U6). No parent had given any reasons against vaccination.

**Table 2.** Characteristics of anti-HBc-positive participants, KiGGS Wave 2, *n* = 7

No.	HBsAg positive	Anti-HBc positive	Anti-HBs positive	Sex	Age group	Migration background	Number of hepatitis B vaccine doses
1	No	Yes	No	Male	11–13	None	0
2	No	Yes	Yes*	Female	7–10	Two sided	4
3	No	Yes	Yes*	Male	14–17	One sided	4
4	No	Yes	Yes*	Female	14–17	Two sided	Unknown
5	No	Yes	Yes	Male	11–13	Unknown	Unknown
6	No	Yes	Yes	Female	7–10	None	4
7	No	Yes	Yes	Female	14–17	None	Unknown

* Anti-HBs positive at re-testing (2.00 < 10.00 mIU/mL).

Four participants had presented their vaccination cards at the time of data collection. One had not received an HBV vaccination dose. Three participants had received all four recommended HBV vaccinations (Table 2). All three vaccinated participants had received their first dose more than 30 days after the day of birth and all had more than 5 months between the third and the fourth doses of HBV vaccination. Two participant had received four doses of Infanrix Hexa. One participant had received one dose of Infanrix Hexa and three doses of Hexavac. Univariable analysis did not show any statistical differences in socio-economic factors, migrant status, and health characteristics reported in the questionnaires and being anti-HBc positive or not. However, the low prevalence meant that the sample size was insufficient to reliably detect any potential differences (results not shown).

## Discussion

This is the second and latest nationwide population based sero-survey measuring hepatitis B prevalence among children and adolescents in Germany. Our results support the evidence that Germany has reached the WHO Euro target of ≤0.1% HBsAg positive in cohorts born after the introduction of universal childhood hepatitis B vaccination in 1995. We found no simultaneous HBsAg and anti-HBc-positive participants and a very low prevalence of anti-HBc in children and adolescents in Germany. All seven anti-HBc-positive participants were born before 2008. Due to the low prevalence, we were not able to investigate characteristics associated with being anti-HBc positive further.

Our results correspond well with those from other low prevalence countries in Europe. The Netherlands reported only two HBsAg positives and a population prevalence of anti-HBc of 0.5% (95% CI: 0.2–1.1) in 0- to 14-year-olds in a population based survey in 2007 [23]. In addition, a sero-survey in Moldova also found a nationwide HBsAg prevalence of 0.21% (95% CI: 0.08–0.53) in children born in 2013 [24]. This supports the assumption that prevention efforts against HBV transmission are efficient in countries with universal childhood vaccination programmes and that mother-to-child transmission interventions such as screening during pregnancy with post-exposure prophylaxis or birth-dose vaccination are successful.

With an estimated national anti-HBc prevalence of 0.3% (95% CI: 0.1–0.8) among children and adolescents in Germany, and no serological results indicating participants with active or chronic infections, our study corroborates previous evidence of a very low prevalence of HBV infections in the general population in Germany [12–15]. When we compared the prevalence found in KiGGS Wave 2 to the findings in KiGGS Baseline, we were not able to detect a significant difference in anti-HBc prevalence between the two surveys. The higher level of variation to the mean for our estimate and the larger 95% confidence interval suggest that larger sample sizes may have been necessary to demonstrate any prevalence differences between the two time periods (Supplementary Table S1).

The low prevalence and lack of anti-HBc- and HBsAg-positive participants in age group 3–6 years confirms the success of hepatitis B prevention programmes in Germany. Although a comparison with KiGGS Baseline is limited due to lack of power, 0.2% (95% CI: 0.0–0.3) of participants aged 3–6 years were found to be anti-HBc positive in KiGGS Baseline [15]. Given the lower mean age and larger proportion of excluded participants being 3–6 years old, we cannot exclude that our finding is due to under-ascertainment and thus underestimation of the prevalence in this age group.

Unlike previous studies [12, 13, 15], we were unable to investigate migration background as a risk or predictive factor for hepatitis B infection for children and adolescents in Germany. This is due to the low number of anti-HBc- and HBsAg-positive participants leading to lack of power needed to detect any association there might be present. Despite the large sample size, this also applies to the assessment of possible associations between other socio-demographic and health characteristics, for example, vaccination status and being anti-HBc positive. A second reason could be under-representation of migrants from high-prevalence countries. In our study, a larger proportion of excluded participants were migrants in second or higher generation compared to included participants; in particular, within participants with a one-sided migration background (results not shown). We cannot exclude an under-ascertainment and thus an underestimation of the prevalence in people with migration background.

The success of hepatitis B prevention programmes in Germany is also supported by other national studies. First, of all pregnant women in 2011–2015, 91.6% were screened for hepatitis B infection and post-exposure prophylaxis was offered on indication [9]. Second, HBV vaccination coverage in Germany increased after the introduction of a polyvalent vaccine, but remained relatively constant between 84% and 88% since 2010 [11]. Finally, using routine surveillance data, a mean incidence of probable mother-to-child transmission cases in Germany was found to be 0.05/100000 [25]. The authors assessed that more data are still needed to assess mother-to-child transmission rate [25], which is a second WHO target to measure impact of elimination of mother-to-child transmission of HBV. The WHO target is a mother-to-child transmission rate of ≤2%, when a country, like Germany, use a targeted birth dose after HBV screening in pregnancy [5–8].

### HBsAg-positive cases

Our identification of three participants who were HBsAg and anti-HBs positive as well as anti-HBc and PCR negative may be a result of the testing strategy. HBsAg- and anti-HBs-positive only participants are rarely reported in sero-surveys. In KiGGS Wave 2, all participants were tested for HBsAg, anti-HBc, and anti-HBs, whereas in KiGGS Baseline only anti-HBc-positive participants were tested for HBsAg. Only testing anti-HBc-positive individuals for HBsAg, and not testing for anti-HBs, is reported in several HBV sero-surveys [23, 24, 26]. The testing strategy may be due to the scope of these surveys and may have been influenced by the combination sero-markers that are naturally occurring and well documented. In addition, the feasibility and extensive costs of testing all participants for HBsAg in a national survey are important aspects. These sero-surveys, like KiGGS Baseline, will thus not be able to identify participants who are only HBsAg and anti-HBs positive. Consequently, these HBsAg and anti-HBs positive only participants are rarely reported in sero-surveys.

The expert opinion on the three HBsAg-positive participants was that the, for sero-surveys, rare finding may be due to the vaccine antigen still being detectable. One participant with anti-HBs >1000 mUI/mL was assessed as having protective vaccination status and two of the three participants with anti-HBs <100 mUI/mL were interpreted as having below protective level of antibodies after vaccination. The three participants had each received four doses of HBV vaccine.

### Overall limitations

The findings of our study are subject to overall limitations due to the study design and low prevalence. First, our study is a cross-sectional study and can therefore only provide a snapshot of the status of HBV prevalence without indicating the time of infection. This means that we are not able to interpret the causality, especially with regard to the date of vaccination. However, in combination with other studies, we still obtain a good indication of the success of the preventive interventions. Second, our study population includes 3- to 17-year-old children and adolescents, and our sample size calculation thus based on an expected prevalence in these vaccinated birth cohorts. However, the WHO target is set for children 0–4 year of age. Third, although the sensitivity and specificity of the laboratory tests are high according to the producer, the positive predictive value will be low due to the low prevalence, which may lead to some degree of misclassification. We defined the level of anti-HBs ≥10 mIU/mL as positive, which may lead to misclassification and under-reporting of anti-HBs-positive participants, as detection may happen on a lower level. In particular, the possible misclassification may affect the 3000 anti-HBc-negative participants, as we did not re-test them. However, this does not change our main findings and the reported overall HBV prevalence. Third, we re-tested all 10 anti-HBc- and HBs-Ag-positive samples at the National Reference Centre for Hepatitis B and D Viruses, enabling us to confirm our findings, which was important given the low prevalence. However, we also may have introduced a bias towards under-reporting, within the anti-HBc-negative participants, as re-testing all samples may have identified more HBs-Ag-, anti-HBc-, and anti-HBs-positive participants. Re-testing all samples was unfortunately not feasible within the scope of this study. Finally, data on socio-demographic and health characteristics, such as participation in recommended health examinations, are self-reported and are subject to the limitations of such survey data. We have tried to address any misclassification due to recording errors by conducting quality checks for participants with implausible vaccination dates or with a first dose given prior to 2 months of age.

## Conclusion and recommendations

Using the cross-sectional data collected during the second wave (2014–2017) of the nationwide representative sero-survey, KiGGS, we demonstrated that the prevalence of viral hepatitis B in cohorts born after introduction of viral hepatitis B vaccination is very low in Germany. This corroborates the body of evidence that Germany is on its way to eliminate hepatitis B as a public health threat. Maintaining high coverage of hepatitis B vaccination among children, together with other mother-to-child transmission prevention measures such as anti-natal screening and post-exposure prophylaxis is, however, necessary to sustain the reached impact.

## Supporting information

10.1017/S0950268825100563.sm001 Gillesberg Lassen et al. supplementary material 1 Gillesberg Lassen et al. supplementary material

10.1017/S0950268825100563.sm002 Gillesberg Lassen et al. supplementary material 2 Gillesberg Lassen et al. supplementary material

## Data Availability

The authors confirm that some access restrictions apply to the data underlying the findings. The dataset cannot be made publicly available because informed consent from study participants did not cover public deposition of data. However, the minimal dataset underlying the findings is archived in the ’Health Monitoring’ Research Data Centre at the Robert Koch Institute (RKI) and can be accessed by all interested researchers. On-site access to the dataset is possible at the Secure Data Center of the RKI’s ’Health Monitoring’ Research Data Centre. Requests should be submitted to the Research Data Centre, Robert Koch Institute, Berlin, Germany (e-mail: fdz@rki.de).

## Supplementary material

The supplementary material for this article can be found at http://doi.org/10.1017/S0950268825100563.
