# Supplementary material for: Evidence of very low hepatitis B virus prevalence in children and adolescents in Germany: National cross-sectional study, 2014–2017
Source: Epidemiol Infect. 2025 Sep 26;153:e120. doi: 10.1017/S0950268825100563 (PMC12529418; doi:10.1017/S0950268825100563)
Supplement: Gillesberg Lassen et al. supplementary material 1 — Gillesberg Lassen et al. supplementary material [file S0950268825100563sup001.docx]

Supplementary Table 1: Weighted prevalence estimates of anti-HBc positive children and adolescents in Germany using data from KiGGS Baseline (2003-2006) and KiGGS Wave 2 (2014-2017), respectably, along with their 95% confidence intervals and variations to the mean (CV)

|  | **2003-2006** | **2014-2017** |
| --- | --- | --- |
| Weighted estimate, Proportion anti-HBc+ | 0.5% [95% CI: 0.4%-0.6%]^a)^ | 0.3% [95%CI: 0.1% - 0.8%] |
| CV | 15% | 49% |

^A)^ Originally reported by Cai et al, 2011: Hepatitis B Virus Infections Among Children and Adolescents in Germany – Migration Background as a Risk factor in a Low Seroprevalence Population and recalculated in subanalysis with weight for comparison with KiGGS Wave 2
