# Supplementary material for: Evidence of very low hepatitis B virus prevalence in children and adolescents in Germany: National cross-sectional study, 2014–2017
Source: Epidemiol Infect. 2025 Sep 26;153:e120. doi: 10.1017/S0950268825100563 (PMC12529418; doi:10.1017/S0950268825100563)
Supplement: Gillesberg Lassen et al. supplementary material 2 — Gillesberg Lassen et al. supplementary material [file S0950268825100563sup002.docx]

Supplementary Table 2: non-weighted distribution of study population characteristics by inclusion/exclusion, KiGGS Wave 2, Germany, 2014-2017, N=3567

| **Characteristics** | | **Total participants in examination part**  **N=3567** | **Included participants, N=3007 (84%)** | **Excluded participants, N=560 (16%)** |
| --- | --- | --- | --- | --- |
| **Age (years), median (IQR)** | | 10 (7-14) | 11 (7-14) | 7 (4-10) |
|  | | **n (%)** | **n (%)** | **n (%)** |
| **Age group (years)** | 3-6 | 885 (25) | 625 (21) | 260 (46) |
|  | 7-10 | 911 (26) | 744 (25) | 167 (30) |
|  | 11-13 | 816 (23) | 733 (24) | 83 (15) |
|  | 14-17 | 955 (27) | 905 (30) | 50 (8.9) |
| **Sex** | Male | 1,766 (50) | 1,485 (49) | 281 (50) |
|  | Female | 1,801 (50) | 1,522 (51) | 279 (50) |
| **Birth-cohort** | 1994-1997 | 66 (1.9) | 62 (2) | 4 (0.7) |
|  | 1998-2001 | 919 (26) | 864 (29) | 55 (9.8) |
|  | 2002-2005 | 1016 (28) | 901 (30) | 115 (21) |
|  | 2006-2009 | 934 (26) | 736 (24) | 198 (35) |
|  | 2010-2013 | 630 (18) | 442 (15) | 188 (34) |
|  | 2014-2017 | 2 (0.1) | 2 (0.1) | 0 (0.0) |
| **Geographical place of living** | Eastern Germany (incl. Berlin) | 1229 (34) | 1,021 (34) | 208 (37) |
|  | Western Germany | 2338 (66) | 1986 (66) | 352 (63) |
| **Municipality size** | Rural; <5,000 inhabitants | 707 (20) | 617 (21) | 90 (16) |
|  | Small town; 5,000<20,000 | 1042 (29) | 864 (29) | 178 (32) |
|  | Middle sized town; 20,000<100,000 inhabitants | 1011 (28) | 839 (28) | 172 (31) |
|  | Urban; >=100,000 inhabitants | 807 (23) | 687 (23) | 120 (21) |
| **Socio-economic status** | Low | 531 (15) | 424 (14) | 107 (19) |
|  | Middle | 2111 (59) | 1781 (59) | 330 (59) |
|  | High | 798 (22) | 696 (23) | 102 (18) |
|  | Missing | 127 (3.6) | 106 (3.5) | 21 (3.8) |
| **Migration status** | None | 2640 (74) | 2244 (75) | 396 (71) |
|  | One-sided | 333 (9.3) | 266 (8.9) | 67 (12) |
|  | Two-sided | 496 (14) | 412 (14) | 84 (15) |
|  | Missing | 98 (2.8) | 85 (2.8) | 13 (2.3) |
| **Migration generation** | First generation | 110 (3.1) | 94 (3.1) | 16 (2.9) |
|  | Second or more generations | 719 (20) | 584 (19) | 135 (24) |
|  | Missing or non-applicable | 2738 (77) | 2329 (77) | 409 (73) |
| **Level of education, CASMIN classification (maximum of the parents in the household) [21]** | Low level | 404 (11) | 319 (11) | 85 (15) |
|  | Middle level | 1806 (51) | 1519 (51) | 287 (51) |
|  | Higher level | 1216 (34) | 1049 (35) | 167 (30) |
|  | Missing | 141 (4.0) | 120 (4.0) | 21 (3.8) |
